# How can surgical skills in laparoscopic colon surgery be objectively assessed?—a scoping review

**DOI:** 10.1007/s00464-021-08914-z

**Published:** 2021-12-06

**Authors:** Tora Rydtun Haug, Mai-Britt Worm Ørntoft, Danilo Miskovic, Lene Hjerrild Iversen, Søren Paaske Johnsen, Anders Husted Madsen

**Affiliations:** 1 Department of Surgery, Regional Hospital West Jutland, Herning, Denmark; 2 Aarhus University, Aarhus, Denmark; 3 Department of Surgery, Aarhus University Hospital, Aarhus, Denmark; 4 St Mark’s Hospital, Northwick Park, Harrow, England; 5 Danish Center for Clinical Health Services Research, Department of Clinical Medicine, Aalborg University, Aalborg, Denmark

**Keywords:** Technical skills, Assessment tool, Competency, Surgical education, Laparoscopy, Colon surgery

## Abstract

**Background:**

In laparoscopic colorectal surgery, higher technical skills have been associated with improved patient outcome. With the growing interest in laparoscopic techniques, pressure on surgeons and certifying bodies is mounting to ensure that operative procedures are performed safely and efficiently. The aim of the present review was to comprehensively identify tools for skill assessment in laparoscopic colon surgery and to assess their validity as reported in the literature.

**Methods:**

A systematic search was conducted in EMBASE and PubMed/MEDLINE in May 2021 to identify studies examining technical skills assessment tools in laparoscopic colon surgery. Available information on validity evidence (*content*, *response process*, *internal structure*, *relation to other variables,* and *consequences*) was evaluated for all included tools.

**Results:**

Fourteen assessment tools were identified, of which most were procedure-specific and video-based. Most tools reported moderate validity evidence. Commonly not reported were rater training, assessment correlation with variables other than training level, and validity reproducibility and reliability in external educational settings.

**Conclusion:**

The results of this review show that several tools are available for evaluation of laparoscopic colon cancer surgery, but few authors present substantial validity **f**or tool development and use. As we move towards the implementation of new techniques in laparoscopic colon surgery, it is imperative to establish validity before surgical skill assessment tools can be applied to new procedures and settings. Therefore, future studies ought to examine different aspects of tool validity, especially correlation with other variables, such as patient morbidity and pathological reports, which impact patient survival.

**Supplementary Information:**

The online version contains supplementary material available at 10.1007/s00464-021-08914-z.

Minimally invasive surgery has increasingly become standard of care in many fields of colorectal surgery. The assessment of the surgeons’ operative performance is highly relevant for quality assurance, training, and certification; it has been shown that technical skill scores vary significantly, even amongst experienced surgeons, and predict the likelihood of adverse clinical outcomes [1–3]. Prior results showed that the variation in the surgeons’ technical skills scored by an observational tool was directly related to the variation in patient complications [2]. Therefore, measures to identify individuals that require further training, to highlight specific training needs, and to define areas of improvement are desirable but often lacking in the clinical setting.

A range of tools to objectively assess surgical performance have been developed and validated in most surgical specialties. They can be divided into three main categories: global rating scales (GRS), procedure-specific tools (PST) and error-based rating scales (ERS). The GRS aim to assess general aspects of the technical expertise and can be applied across surgical procedures [4–6]. The most cited and widely used tool in this category is the Objective Structured Assessment of Technical Skill (OSATS), developed by Martin et al. in 1997 [6]. GRS are reliable and valid for numerous procedures, but they do not provide feedback on a specific step or a particular technique. PST are dedicated to a single specific procedure and each step or task area of an operation can be individually rated [7]. ERS aim to identify errors and near misses as a surrogate for the overall quality of the performance [8]. Analysis of error types or errors performed during parts of the procedure can give a detailed insight into skill or procedure specific areas that need further development.

Laparoscopic colorectal surgery and other minimally invasive techniques require some of the most complex skills in general surgery [9]. Especially in colon and rectum cancer surgery, surgical precision and completeness of the resection margins are highly relevant. The completeness of the mesorectal or mesocolic excision has been associated with reduced cancer recurrence rates and highlights the fragile relationship between surgical skill and patient outcome [10–12]. In such high-stake surgical environments, the use of objective formative and summative assessment during training and beyond is highly relevant for quality assurance. Although there is evidence of reliable and valid assessment tools, clinical implementation of tools for the assessment of operative quality in especially laparoscopic colon surgery is sparse. Also, little is known about the validity of such tools, supporting an appropriate interpretation of assessment results [13, 14].

Therefore, the aim of this scoping review is to comprehensively identify tools for skill assessment in laparoscopic colon surgery, and to assess their validity as reported in the literature.

## Material and methods

This scoping review was conducted according to PRISMA guidelines with Extension for Scoping Reviews [15]. As scoping reviews are not included at the systematic reviews database, PROSPERO, the present protocol can be obtained on request to the corresponding author.

### Eligibility criteria

Inclusion criteria were any research study assessing observational tools of technical skills in laparoscopic colon surgery, and the manuscript written in English. Studies performed on virtual reality simulators and studies solely assessing non-technical skills, such as communication skills, teamwork, leadership, and decision-making were excluded. Studies assessing tools for both technical and non-technical evaluations were included in this review. Conference abstracts, reviews, and editorials were excluded. No restrictions to the publication date were imposed.

### Search strategy

The EMBASE and PubMed/MEDLINE databases were used to identify relevant studies, and the Cochrane database was also searched to include any reviews on the subject. All references of the included full-text articles were reviewed to identify studies that might have been overlooked. The PubMed/MEDLINE search was performed using free text words describing competency assessment, colon surgery, and laparoscopy. A combination of the Medical Subject Headings ([MeSH]) terms ‘clinical competence’, ‘colon resection’ and ‘laparoscopy’ was used. A similar search strategy was applied to EMBASE, though with modification as needed. The final search was performed on the 28^th^ of May 2021 and the search string of use is presented in Supplemental Table 1.

**Table 1:** Definitions of validity sources. Adopted from Beckman et al. [16] and Ghaderi et al. [13] Modified for the scope of this review

Domain	Definition	Score	Description	Examples
**Content validity**	The extent to which the tool’s content relates to the construct it intends to measure	0	No data regarding the developing process	
		1	Expert judgment with limited data regarding the tool content	Expert judgment
		2	Listing assessment items for the tool content with some references to a panel of experts, limited description of the developing process	Structured task analysis, hierarchical task analysis
			References to a previously validated tool	Based on previously validated tools
		3	Well-defined developing process, both theoretical basis for the chosen items and systematic review by experts	Delphi-method, pilot study
**Response process**	The analysis of the responses given by the individual assessors and interpretation of the reported results	0	No data regarding the response process	
		1	Limited data reported. Use of an assessment tool without discussing the impact of the differences in response processes	User manuals
		2	Some data regarding different responses of assessors. Some data about systems that reduce variation between respondents	Structured assessor training before the assessment process
		3	Multiple sources of data examining response error through critical examination of response processes and respondents	Validation of initial scores (pilot study), evaluation of response error after structured assessor training
**Internal structure**	The extent to which individual items describe the underlying constructs, often reported by measures of inter-rater reliability, internal consistency and generalizability	0	No data regarding internal structure	
		1	Limited data regarding internal structure, references to a single inter-rater reliability measure	Simple measures of inter-rater reliability (ICC or Cronbach alpha) *or* interitem-reliability
		2	A few measures of reliability reported, insufficiently item analysis	Inter-rater reliability coefficient combined with a single measure of interitem or intertest reliability
		3	Multiple measures of reliability including inter-rater reliability and item-analysis (interitem reliability, inter-test reliability, item response theory)	Generalizability theory analysis, item response theory
**Relations to other variables**	Correlation between the assessment scores and other outcomes or scoring systems relevant to the construct being measured	0	No data regarding relations to other variables	
		1	Correlation of scores to outcomes with limited theoretical importance, references to a single measure of validity	Compare level of training to score achieved with the assessment tool
		2	Correlation of scores to outcomes with theoretical importance, references to a few measures of validity	Correlation with level of training and clinical data (operative time, patient outcomes etc.)
		3	Correlation or no correlation between important theoretical outcomes or scores of the same construct	Correlation with training level, clinical data and other performance assessment tools, generalizability evidence
**Consequences**	The impact of the assessment tool and future use	0	No data or discussion regarding consequences	
		1	Limited data, merely a discussion about future use	Describing feasibility and potential future use (data on assessment time, post assessment survey)
		2	Some descriptions of consequences of assessment for learners, often supported by incomplete data	Describing educational impact (formative / summative feedback, learning curve of trainees)
		3	Clear description of consequences of assessments and the impact on interpretation of scores and intended future use, supported by data	Criterion-referenced score (pass/fail-scores), cut-of scores for licensing purposes, predictive models

### Study selection

All studies examining assessment tools of technical skills in laparoscopic colon surgery were included. Assessment tools were defined as a blinded or non-blinded assessment of technical skills performed live or on video, based on pre-defined rating criteria. Step-by-step descriptions of procedures were excluded if surgical performance was not translated into a summative result on an arbitrary scale. Also not considered were non-observational tools such as dexterity-based systems (e.g. instrumental path length or number of movements) and studies examining technical performance at task-specific stations not considering full-length procedures. The number of procedures or registration of postoperative complications were not considered observational assessments of technical skill.

Further, studies were only considered if the assessment tool described were aimed towards laparoscopic colon procedures: right and sigmoid colectomies, total and subtotal colectomies were all included. Studies examining tools applied to ‘laparoscopic colorectal procedures’ in general, without specifying any further detail, were included in the review. No restrictions were made to the indication of the laparoscopic colonic procedure (benign/malignant) or to the development, validation, or implementation process of the tool. Studies assessing tools solely aimed towards laparoscopic rectal surgery were not considered. Also, tools developed for open colon surgery or robotic colorectal surgery were excluded.

### Data collection and study assessment

All studies were screened individually by two authors (TH, MBO) using the systematic review software Covidence (Veritas Health Innovation, Melbourne). Full-text articles were retrieved for all eligible manuscripts. Details regarding the validation process were extracted separately by the two authors comprising whether the tool was applied to surgical trainees or consultants; the number of assessors; the type of procedures evaluated; video versus live assessment; and the validation setting. The same two authors then rated the included studies for validity evidence according to the score provided by Beckman et al. [16], which later have been broadened by Ghaderi et al. [13]. This scoring system provides a framework of five dimensions of validity: i) content, ii) response process, iii) internal structure, iv) relations to other variables, and v) consequences (Table 1).

In short, *content validity* describes the degree to which the tool’s content measures the construct of interest and refers to the themes, wording, and format of the tool items. The *response process* describes how the assessments given by the individual assessors are analysed. Evidence of *internal structure* refers to the degree to which the tool items fit the underlying constructs, and the *relation to other variables* describes the relationship between the tool scores and external variables e.g. surgeon experience level. Evidence of *consequences* is defined as the intended and unintended impact of the tool use. In the present study, each of these five dimensions was assigned with a score ranging from 0 to 3, for a total score of 15. The total validity score was then graded as follows; 1–5 limited evidence, 6–10 moderate evidence, and 11–15 substantial evidence. The definitions of validity evidence used, with examples of numerical scores, can be found in Table 1. Any disagreement between the two authors regarding study selection, data extraction, or validity evidence was resolved by discussion.

## Results

### Literature search and study selection

**Fig. 1:**
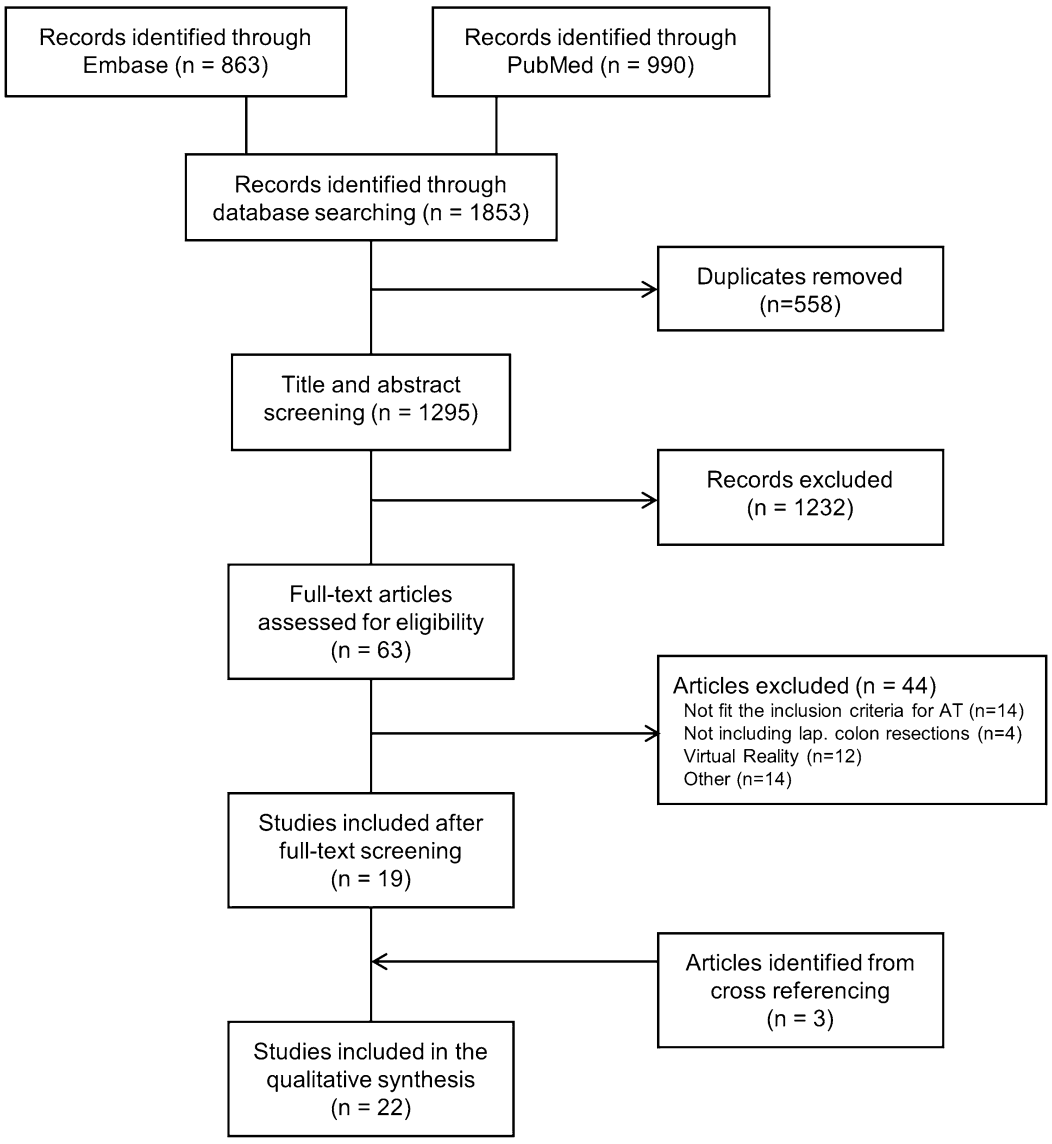
Flowchart of the included studies. AT: assessment tool, lap. colon: laparoscopic colon, other: language, review, protocol paper, editorial, conference abstract, commentary

The study selection process is described in Fig. 1. In short, the primary literature search revealed 1,853 studies. After removing 558 duplicates, the remaining 1,295 titles and abstracts were screened for relevance. Of these, 63 studies underwent a full-text review, of which 19 met the inclusion criteria [1, 2, 7, 8, 17–31]. Three additional studies were included after reviewing full-text references [32–34].

### Characteristics of the assessment tools

The search process identified 22 studies, which presented 14 different tools for technical skill assessment in laparoscopic colon surgery (Table 2). On reviewing the included tools’ contents, the studies were grouped into the three main tool categories: five were GRS [17–20, 32], one was an ERS [8], and eight were PST [22–24, 27, 29–31, 33]. The studies were primarily conducted in the United Kingdom, Canada, the United States, and Japan.

**Table 2:** Characteristics*

Assessment tools	14 (100%)	
*Type of assessment tool*		
Global rating scale	5 (36)	
Error-based rating scale	1 (7)	
Procedure-specific tool	8 (57)	
*Year of publication*		
2005–2010	4 (29)	
2011–2015	6 (43)	
2016–2020	4 (29)	
*Country*		
United Kingdom		5 (36)
Canada		4 (29)
United States		3 (21)
Japan		2 (14)
*Modified or original*		
Modified	5 (36)	
Original	7 (50)	
Modified and original	2 (14)	
*Video or direct observation*		
Video	7 (50)	
Direct observation	5 (36)	
Both video and direct observation	1 (7)	
Unspecified	1 (7)	

^*^A total of 22 studies were identified which included 14 different assessment tools. Only the paper describing the developing process has been included for the tools described in multiple papers

The identified tools included seven original tools, five modified versions of previously validated tools, and two tools that were a combination of these (Table 3). Eleven were evaluated on surgical procedures performed in the operating theatre, two were used in a laboratory setting (animal models) and one provided no setting information (Table 4). Five tools were applied to surgical trainees, four to surgical consultants, and another four tools to a combination of these. Concerning the surgical procedure used for assessment, seven tools were applied to video-recorded cases, five to direct observation, one reported no preferences, and one tool was applicable to both. One assessor per case was reported for all tools using direct observation, whereas two or more assessors were described for tools using video-recorded cases. Use of the assistant was considered in five tools: SAS, OSATS, OCRS, CT and ASLAC*.* A large variation was observed for the surgical cases evaluated in the included studies, ranging from 0 to 750 [19, 31].

**Table 3:** Descriptive data of assessment tools

	Tool		Abb	Year	Items	Versions	Score range	Original or modified
Global Rating Scale								
1		Sidhu et al. [17] *Self-assessment scale*	SAS	2006	11	1	11–55	Previously validated GOALS and OSATS
2		Wohaibi et al. [18] *OpRate*	OpRate	2007	6	1	1–4 (m)	Original
3		Niitsu et al. [32] *OSATS*	OSATS	2012	7	1	7–35	Previously validated OSATS
4		Jenkins et al. [19] *GMAS/DOPS*	GMAS	2016	12	1	0–45	Original GMAS and previously validated DOPS
5		Watanabe et al. [20] *IRT-GOALS*	IRT-GOALS	2017	5	1	5–25	Previously validated GOALS
Error-based rating scale								
6		Miskovic et al. [8] *OCHRA*	OCHRA	2012	3	1	3–25	Previously validated GAS, and OCHRA
Procedure-specific tool								
7		Dath. et al. [33] *OCRS*	OCRS	2003	7	1	1–5(m)	Original OCRS and previously validated OSATS
8		Sarker et al. [21, 22]* *Technical skills assessment tool for laparoscopic colectomy*	TSALC	2010–2011	7–9	3^%^	35–45	Original
9		Palter et al. [7, 23]* *Procedure-specific evaluation tools*	PSET	2011–2012	18 or 18/19	2 ^&^	50–80 or 50–90	Original
10		Miskovic et al. [24–26, 34]* *GAS-tool*	GAS	2011	12	1	1–72	Original
11		Miskovic et al. [1, 27, 28]* *CAT-tool*	CAT	2013	16	1	1–4 (m)	Original
12		Glarner et al. [29] *Comprehensive assessment Tool*	CT	2013	8	1	1–5 (m)	Original CT and modified OSATS and NOTSS
13		Champagne et al. [2, 30]* *ASCRS Tool*	ASCRS	2017	9	1	1–5 (m)	Previously validated OSATS and OCHRA
14		Nakayama et al. [31] ASLAC score	ASLAC	2020	35	1	NR	Original

*Abb:* Abbreviation *Year*: year of publication *Items*: number of statements evaluated on a rating scale. If the tool includes general, non-technical and procedure-specific items, only the procedure-specific items will be considered *Versions*: number of versions available for laparoscopic colon procedures *NR*: not reported *(m)* mean

^*^Only number from the first validation paper has been reported

**Table 4:** Data describing the validation process of assessment tools

	Tool	Partici-pants	Cases†	Procedure evaluated	Validation setting	Live / video	Assessor per case	Assessors in total
Global Rating Scale								
1	Sidhu et al. [17] *Self-assessment scale*	22 (T)	22	Lap sigmoid colectomy	Laboratory (animal)	video	2	2
2	Wohaibi et al. [18] *OpRate*	29 (T)	579†	Lap colon resections †	Workplace	live	1	33
3	Niitsu et al. [32] *OSATS*	10 (T)	757†	Lap colon resections †	Workplace	live	1	12
4	Jenkins et al. [19] *GMAS/DOPS*	8 (C)	750	Lap colon and rectum resections	Workplace	video, live	1	2
5	Watanabe et. al [20] *IRT-GOALS*	396 (T + C)	396†	Lap colon and rectum resections †	Workplace	live	1	NR
Error-based rating scale								
6	Miskovic et al. [8] *OCHRA*	21 (C)	33	Lap colon resections (right and left colectomies)	Workplace	video	1–3 (2 for sum-mative feedback)	16
Procedure-specific tool								
7	Dath. et al. [33] *OCRS*	29 (T)	58†	Lap low anterior resections	Laboratory (animal)	video	2	10
8	Sarker et al. [21, 22]* *Technical skills assessment tool for laparoscopic colectomy*	14 (T + C)	84	Lap colon and rectum resections (right hemicolectomies, sigmoid resections and anterior resections)	Workplace	video	2	2
9	Palter et al. [7, 23]* *Procedure-specific evaluation tools*	37 (T + C)	37	Lap right colectomies and lap sigmoid colectomies	Workplace	video	2	2
10	Miskovic et al. [24–26, 34]* *GAS-tool*	52 (C)	333	Lap colon and rectum resections (right hemicolectomies, sigmoid resections, anterior resections, low anterior resection, total and subtotal colectomies and assisted abdominoperineal resections)	Workplace	live	1–2 (self-evaluation by subject)	30
11	Miskovic et al. [1, 27, 28]* *CAT-tool*	31 (C)	54	Lap right and left colectomies	Workplace	video	2–3	27
12	Glarner et al. [29] *Comprehensive assessment Tool*	16 (T)	63	Lap segmental colon resections	Workplace	live	1	4
13	Champagne et al. [2, 30]* *ASCRS Tool*	24 (T + C)	24	Lap right hemicolectomies	Workplace,	video	5	20
14	Nakayama et al. [31] ASLAC score	NR	NR	NR	NR	NR	NR	NR

Participants: number of participating trainees (T) and consultants (C) Trainees: doctors purchasing a career in surgery who have not completed their residency as post-graduate year surgeons Consultants: surgeons who have completed their post-graduate residency and those who are specialised in operating on colon and rectum Cases: number of live or video recorded operations included in the analysis Lap: laparoscopic Assessors pr. case: number of assessor on each procedure Assessors in total: assessors contributing to the scoring process NR: not reported

^†^Other surgical procedures also reported

^*^Only number from the first validation paper has been reported

### Evaluation of validity evidence

All tools were scored according to *content*, *response process*, *internal structure*, *relations to other variables*, and *consequences*, as exemplified in Table 1. The validity evidence score for all assessment tools is presented in Table 5.

**Table 5:** Evidence of validity

	Tool	Content	Response process	Internal structure	Relations to other variables	Conse-quences	Total
Global Rating Scale							
1	Sidhu et al. [17] *Self-assessment scale*	2	1	2	1	1	**7**
2	Wohaibi et al. [18] *OpRate*	0	1	1	1	0	**3**
3	Niitsu et al. [32] *OSATS*	2	2	0	1	1	**6**
4	Jenkins et al. [19] *GMAS and modified DOPS (GMAS)*	2	2	0	2	3	**9**
5	Watanabe et al.[ 20] *IRT-GOALS*	2	0	3	1	3	**9**
Error-based rating scale							
6	Miskovic et al. [8] *OCHRA*	2	2	2	3	3	**12**
Procedure-specific tool							
7	Dath. et al. [33] OCRS	2	1	2	1	1	**7**
8	Sarker et al. [21, 22]* *Technical skills assessment tool for laparoscopic colectomy (TSALC)*	2	0	1	1	1	**5**
9	Palter et al. [7, 23]* *Procedure-specific evaluation tools (PSET)*	3	1	1	1	1	**7**
10	Miskovic et al. [24–26, 34]* *GAS-tool*	2	1	2	1	3	**9**
11	Miskovic et al. [1, 27, 28]* *CAT-tool*	3	1	3	3	3	**13**
12	Glarner et al. [29] *Comprehensive assessment Tool (CT)*	3	1	0	1	1	**6**
13	Champagne et al. [2, 30]* *ASCRS Tool*	3	2	2	2	3	**12**
14	Nakayama et al. [31] ASLAC score	3	0	0	0	0	**3**

^*^Scoring system: 0: the study provided no discussion or data, 1: the study provided limited data that support validity evidence, 2: the study provided some data (intermediate) that support validity evidence, 3: the study provided multiple data that support validity evidence

^*^The highest level of validity for the respective studies is reported

#### Content

The evidence of content validity varied across the tool categories (score 0–3). Eight studies provided moderate evidence (score 2) as these relied on previously validated tools or a combination of an original and a previously validated tool [8, 17, 19, 20, 22, 24, 32, 33]. Of these, three were modified versions of the OSATS [6]. Task analyses based on textbooks, articles, video recordings, and expert discussions was used to create the tool of Sarker et al. (TSALC) [22] and the GAS of Miskovic et al. [24]. More comprehensive methods that included systematic expert review (Delphi method) were used to establish content validity for the tools of Palter et al.(PSET) [7, 23], Miskovic et al. (CAT) [27], and Nakayama et. al [31]. In line with this, a consensus-achieving method was applied by Champagne et al. (ASCRS) [30], where a panel of experts modified previously validated tools by watching video-recorded laparoscopic right colectomies. Comprehensive methods supporting content validity could also be found in the paper by Glarner et al. [29], where the tool was piloted in the operating room and revised through an iterative process until the researchers and colon surgeons reached consensus. Oppositely, the tool by Wohaibi et al. (OpRate) [18] presented the lowest evidence (score 0), as this paper did not reveal how the content was chosen.

#### Response process

The evidence for the response process validity varied across all studies from 0 to 2. Some studies reported that a brief orientation was given to the assessors (Sidhu et al. (SAS) [17], Dath et al. (OCRS) [33], OpRate, PSET, and CAT) to obtain assessment consistency; others provided no information regarding the response process (Watanabe et al.(IRT-GOALS) [20] and the TSALC).

Structured training of the assessors before initiating the assessment process was reported by four studies, including the paper of Niitsu et al. (OSATS) [32], Miskovic et al. (OCHRA) [8], the Jenkins et al. (GMAS) [19], and the ASCRS studies. Although the ASCRS underwent modification in a pilot phase until the experts reached agreement, the assessors were not evaluated after they had completed rater training, which is why the ASCRS was graded with a moderate level of validity evidence. The GMAS exceeded others by reporting continuous training of the assessors during the study period, although no data was provided regarding the impact of the rater training. None of the tools reported multiple sources of data examining the response process (score 3).

#### Internal structure

The most common reported evidence of internal structure was inter-rater reliability, which was reported by seven tools (50%) [8, 17, 22–24, 30, 33]. No consistent method of calculating inter-rater reliability was used, and the strategies included interclass correlation coefficient, AC1 Gwet coefficient, Pearlson correlation, and Cronbach’s α. OCHRA was the only tool to report test–retest reliability, comparing error counts in cases performed by the same surgeon.

Six studies reported item analysis: internal consistency (inter-item reliability) was described for SAS, OpRate, GAS, PSET, and ASCRS; task-to-task variation (inter-station reliability) was analysed for OCRS.

The IRT-GOALS and CAT were the only tools for which extended measures of inter-item reliability was reported (score 3): Item response theory was used for the IRT-GOALS, and the reliability coefficient of generalizability theory was used for the CAT, examining the effect of an increasing number of assessors and cases by applying the D-studies.

#### Relations to other variables

The evaluation of this dimension revealed that most studies provided either poor (score 0–1) or excellent validity evidence (score 3). Nine studies (64%) compared performance scores across training levels or case experience; all reported improved scores with increased training levels or greater case experience. Comparison with other assessment modalities was described for three tools: GMAS was compared to Direct Observation of Procedural Skills scores; OCHRA was compared to an overall “pass/fail” global score, operating time, and a measure of efficiency (dissecting-exposure ratio); and CAT was compared to an overall outcome statement (fail/pass) as well as OCHRA error counts. Finally, the relationship between assessment tool scores and patient outcomes was examined for CAT and ASCRS, both reporting reduced risks of postoperative morbidity for high-skill level surgeons. Correlation to pathological examination was reported for CAT only, describing less lymph nodes harvested and a shorter distal resection margin for low-skill level surgeons [1].

#### Consequences

In line with *Relations to other variables*, the validity evidence revealed for the *consequences* of the presented assessment tools was either low (score 0–1) or high (score 3).

Four studies reported data regarding ‘time to complete the assessment tool’ [24, 29, 30, 33], whereas three studies describes implementation of the assessment tool in a clinical surgical training programs: GMAS was used in the multimodal training program at St. Mark’s Hospital in London (2006–2010), and GAS/CAT were used in the National Training Program for consultant surgeons in England (2008–2009/2010–2012). While GMAS and GAS were used to provide formative feedback, CAT was used for summative assessment reporting a cut-off score of 2.7 differing between ‘pass’ and ‘fail’ surgeons. The educational impact of the tool score was clearly described for GAS, reporting the number of surgical cases required before trainees felt confident in performing a surgical procedure independently (proficiency gain curve). Likewise, score accuracy was established for CAT and OCHRA using prediction models. Although not officially included in a national surgical education program, also the IRT-GOALS study provided a clear description of the impact of clinical implementation with interpretation of assessment scores using item response theory results.

## Discussion

This scoping review identified 14 tools for skill assessment in laparoscopic colon surgery and described their characteristics and validity. Most of the tools were evaluated in small studies with fewer than 30 participating trainees and 90 operative cases.

A majority of the identified tools were procedure-specific, which reflect the technical complexity of laparoscopic colon surgery, as most surgeons would be expected to have mastered generic laparoscopic skills before embarking on laparoscopic colon resection surgery. Interestingly, side-specific versions were only available for two tools, although it is well known that right and sigmoid colectomies differ considerably in technical complexity. Therefore, for one-version tools, mastering of a complex procedural step, e.g. vascular dissection during a right hemicolectomy, might not be correctly evaluated. As a result, the one-version tool design challenges the content validity (how the tool content relates to the construct it intends to measure). However, it should be emphasised that most of the one-version tools included evaluation of both right and left-sided procedures when results were correlated to other relevant outcomes.

The assessment was predominantly based on video-recorded cases which offers the advantage of multiple assessors evaluating the same procedure at a chosen time. In addition, the independent scoring allows assessors to rewind a surgical step for repeated viewing and to be blinded to the surgeon’s identity and training level, rendering a more objective assessment. On the other hand, video-based assessment can be time consuming. A possible future solution could be the use of artificial intelligence to automatically identify key steps and operative actions, as suggested by Kitaguchi et. al for laparoscopic hemicoletomies [35]. A further limitation of video-based assessments from a purely laparoscopic view are the lack of an external view and audio to assess technical and non-technical skills. As the operating table and theatre are not recorded, the amount of required supervision and support cannot easily be assessed.

The expertise of the assistant was only considered by five tools in this review. Especially during laparoscopic colonic procedures, the tissue exposure relies heavily on the first assistant. Poor technical skills in camera navigation can cause prolonged operating time and increased frustration of the operating surgeon and decrease the quality of the submitted video for skill evaluation. It is obvious that the use of first assistants should be considered when surgical performance is evaluated, as it is the operating surgeons’ ultimate responsibility to always secure excellent exposure. However, the deliberate use of the assistant can be hard to assess watching video-recorded procedures, so it might be more appropriate to include this aspect when evaluating non-technical skills such as leadership and communication. Another possibility would be to adjust for poor camera navigation in the evaluation of surgical performance, due to the laparoscopic camera navigation scoring system by Huettl et. al [36]

More technical aspects should also be considered when evaluating the quality of video-recorded procedures. This has recently been addressed by the paper of Celentano et al. presenting the LAParoscopic surgery Video Educational GuidelineS (LAP-VEGaS) [37] as a standard framework for publication and presentation of surgical videos. When education program directors consider implementation of video-based assessments tools, the role and experience of the camera assistant as well as the LAP-VEGaS guidelines could be helpful in standardising the overall video quality for surgeons’ video-recorded procedures.

Overall, most tools in this review were validated in a clinical setting and reported with an average assessment time, as a common acknowledgment of clinical feasibility. Apart from assessment time, Glarner et al. measured feasibility by reporting the percentage of completed assessments [29]. Further, GAS utility was examined through surveys asking assessors about the perceived usefulness of the tool^24^. Similarly, surveys have been proposed to describe acceptability in the clinic, relevance of tool items, and educational impact for a novel tool in laparoscopic rectal cancer surgery (LapTMEpt) [3]. There seems to be broad agreement that the ease of using a tool may play an important role in the implementation process of a novel assessment tool into clinical practice.

In contrast to authors’ consideration of feasibility, none of the included studies evaluated the effect of rater training, which might be due to time constraints, increased cost, obligations to meet physically, or lack of priority. Though it has previously been shown that trained assessors are more comfortable performing direct observation and more stringent in their evaluations compared to not-trained assessors [38], the effect of rater training on assessment procedure is unclear [39–41]. This can be exemplified in the paper of Robertson and colleagues who examined the reliability of four established assessment tools for suturing and knot-tying for trained versus not-trained assessors [40]. In this paper, rater training tended to improve reliability among assessors but the impact on the performance scores was unclear. Therefore, further studies are needed to determine the effect of rater training and clarify how it should be implemented and evaluated.

Another prominent finding was the substantial number of tools which compared assessment scores to training level, often defined according to the postgraduate year (PGY) of the performing surgeon. As PGY simply refers to years of clinical experience, PGY levels do not necessarily reflect the quality of operative performance. The number of *supervised* procedures, and not just the number of procedures performed, has previously been reported to increase performance scores for laparoscopic colorectal surgeons [1]. Following this argument, technical abilities might vary considerably between trainees at the same PGY level. However, even though training level represents a small facet of construct validity, most of the authors made no further attempt to examine possible correlations with other variables. The relationship between assessment scores and patient outcome was examined for only two of the procedure-specific tools: CAT and ASCRS [1, 2]. In both papers, postoperative complications following laparoscopic colectomies were directly associated to the technical skill ass assessed by the tool.

For cancer surgery, the relationship between performance scores and results of pathological examinations are of particular interest, as the plane of surgery has previously been associated with improved patient survival [12]. Dissection performed in the wrong plane, damage to the mesocolon, or inadequate resection margins are all indicators for poor resection quality. Therefore, it would be beneficial to incorporate the specimen quality in future tool assessment criteria, as presented by Curtis et al. [3] for laparoscopic rectal cancer surgery or as in the right hemicolectomy scoring system for specimens by Benz et al. [42]. Although pathological evaluation was not included in the assessment criteria of the present tools, some authors did evaluate the relationship between assessment scores and the pathological specimen examination. This has been illustrated for CAT scores, where low ratings have been associated with a reduced number of harvested lymph nodes and a shorter distal resection margin in the specimen of laparoscopic colorectal surgery [1]. In rectal cancer surgery, a similar positive correlation has been observed between low error frequency described by OCHRA and the correct plane of dissection [43]. In light of the evidence above, it is obvious that well-established validity evidence describing relations to clinical variables is essential in future surgical improvement initiatives.

A limitation applying to most of the included tools in this review was the lacking evidence for the reproducibility of the results. Several of the included tools have been used regularly in educational settings for technical assessment in laparoscopic colon surgery beyond their initial development and validation phase [8, 18, 22–24, 27, 32]. Some of these tools have been validated in other procedures such as laparoscopic rectal surgery, hernia repair, and gynaecological procedures. However, none have specifically evaluated the validity evidence from the initial validation process in a different population of assessors or patients undergoing laparoscopic colon surgery. An assessment tool whose score provides valid inferences in a specific residency program under research conditions may need further evaluation before use at multiple institutions. Depending on the intended use and consequence of the assessment tool, validity should be demonstrated for each setting separately [44].

A single preferred tool for technical skill assessment in laparoscopic colon surgery has not been highlighted. However, we do recommend clinicians and training program directors to consider implementation of tools that are both easy to use and demonstrate well-established validity evidence. From the results of this review, GAS [24], ASCRS [30], and CAT [27] meet these requirements. Moreover, the assessment setting and endpoint should be considered; where e.g. GAS and ASCRS are used for formative evaluations, CAT is validated for summative evaluations. Further, where GAS is validated for live operations, ASCRS is validated for video-recorded procedures. As we move towards implementation of new techniques, such as laparoscopic complete mesocolic excision (CME), the development of a procedure-specific tool is still lacking, as none of the available tools adequately evaluate the most important procedural aspects of this technique.

It is a limitation of the present study that only tools for technical skill assessment were included. In recent years, non-technical skills in surgery have gained wide interest as it is evident that communication, teamwork, leadership, and decision-making are critical procedure-related skills, complementing the surgeons’ technical abilities [45–47]. However, non-technical skill assessment is a major topic in its own right, so to uphold a clear scope for the present review, studies solely examining tools for non-technical skill assessment were excluded in the study selection process. Tools solely aimed towards laparoscopic rectal surgery were not included, as the procedure-specific operative steps in rectal surgery differ too much compared to those in advanced laparoscopic colon surgery. Neither included were tools aimed towards robotic surgery, as the surgical skills required to use a robotic approach were thought to be substantially different from those required to control laparoscopic instruments and in a clinical setting often reserved for the most experienced surgeons. Furthermore, we chose not to include studies performed on virtual reality simulators (VR), although some simulators include laparoscopic colectomy procedures [48]. Even though VRs are effective at improving basic laparoscopic skills, procedure-specific techniques may not be generalised to the operating room as VRs lack tactile feedback and do not reflect the variation in patient anatomy. Finally, it should be emphasised that evidence for reproducibility of the results from Ghaderi et al.’s scoring system is still lacking, although it has been used in reviews describing assessment tools available for other surgical procedures [49, 50].

## Conclusion

In conclusion, several tools are available for evaluation of laparoscopic colon cancer surgery, but few authors present substantial validity for tool development and use. As we move towards the implementation of new techniques in laparoscopic colon surgery, it is imperative to establish validity before surgical skill assessment tools can be applied to new procedures and settings. Therefore, future studies ought to examine different aspects of tool validity, especially correlation with other variables, such as patient morbidity and pathological reports, which impact patient survival.

## Supplementary Information

Below is the link to the electronic supplementary material.

Supplementary file1 (DOCX 16 KB)
