# Supplementary material for: How can surgical skills in laparoscopic colon surgery be objectively assessed?—a scoping review
Source: Surg Endosc. 2021 Dec 6;36(3):1761–74. doi: 10.1007/s00464-021-08914-z (PMC8847271; doi:10.1007/s00464-021-08914-z)
Supplement: Supplementary file 1 — Supplementary file1 (DOCX 16 KB) [file 464_2021_8914_MOESM1_ESM.docx]

**Supplemental material**

| *Supplemental Table 1: Search strategy* | |
| --- | --- |
| **EMBASE** | |
| **Search** | **Terms** |
| #1 | 'clinical competence'/exp |
| #2 | 'task performance'/exp |
| #3 | 'questionnaire'/exp |
| #4 | 'clinical competence':ab,kw,ti |
| #5 | 'educational measurement':ab,kw,ti |
| #6 | 'task performance':ab,kw,ti |
| #7 | 'task analysis':ab,kw,ti |
| #8 | survey*:ab,kw,ti |
| #9 | 'competency assessment':ab,kw,ti |
| #10 | 'technical skill assessment':ab,kw,ti |
| #11 | 'technical skills assessment':ab,kw,ti |
| #12 | tool*:ab,kw,ti |
| #13 | grading*:ab,kw,ti |
| #14 | checklist*:ab,kw,ti |
| #15 | 'check list*':ab,kw,ti |
| #16 | questionnaire*:ab,kw,ti |
| #17 | rating*:ab,kw,ti |
| #18 | 'scoring system':ab,kw,ti |
| #19 | 'classification system':ab,kw,ti |
| #20 | 'objective evaluation':ab,kw,ti |
| #21 | #1 OR #2 OR #3 OR #4 OR #5 OR #6 OR #7 OR #8 OR #9 OR #10 OR #11 OR #12 OR #13 OR #14 OR #15 OR #16 OR #17 OR #18 OR #19 OR #20 |
| #22 | 'colorectal surgery'/exp |
| #23 | 'colorectal surgeon'/exp |
| #24 | 'colon resection'/exp |
| #25 | 'colorectal surgery':ab,kw,ti |
| #26 | 'colorectal surgeon*':ab,kw,ti |
| #27 | 'colon resection':ab,kw,ti |
| #28 | cme:ab,kw,ti |
| #29 | colectomy:ab,kw,ti |
| #30 | 'complete mesocolic excision':ab,kw,ti |
| #31 | lcme:ab,kw,ti |
| #32 | proctocolectomy:ab,kw,ti |
| #33 | #22 OR #23 OR #24 OR #25 OR #26 OR #27 OR #28 OR #29 OR #30 OR #31 OR #32 |
| #34 | 'laparoscopic surgery'/exp |
| #35 | laparoscop*:ab,kw,ti |
| #36 | #31 OR #34 OR #35 |
| #37 | #21 AND #33 AND #36 |
| #38 | #37 AND ('Article'/it OR 'Article in Press'/it OR 'Erratum'/it OR 'Review'/it OR 'Short Survey'/it) |
|  |  |
| **PubMed/MEDLINE** | |
| **Search** | **Terms** |
| #A | ("Clinical Competence"[MeSH Terms] OR "Educational Measurement"[MeSH Terms] OR "Task Performance and Analysis"[MeSH Terms] OR "Surveys and Questionnaires"[MeSH Terms] OR "Clinical Competence"[Text Word] OR "Educational Measurement"[Text Word] OR "task performance"[Text Word] OR "task analysis"[Text Word] OR "survey*"[Text Word] OR "competency assessment"[Text Word] OR "technical skill assessment*"[Text Word] OR "technical skill score*"[Text Word] OR "tool*"[Text Word] OR "grading*"[Text Word] OR "checklist*"[Text Word] OR "check list*"[Text Word] OR "questionnaire*"[Text Word] OR "rating*"[Text Word] OR "scoring system"[Text Word] OR "classification system"[Text Word] OR "objective evaluation"[Text Word]) |
| #B | ("Colorectal Surgery"[MeSH Terms] OR "Colectomy"[MeSH Terms] OR "Colorectal Surgery"[Text Word] OR "Colectomy"[Text Word] OR "colon resection"[Text Word] OR "cme"[Text Word] OR "complete mesocolic excision"[Text Word] OR "lcme"[Text Word] OR "proctocolectomy"[Text Word] OR "colorectal surgeon*"[Text Word]) |
| #C | ("Laparoscopy"[MeSH Terms] OR "laparoscop*"[Text Word] OR "lcme"[Text Word]) |
| #D | ("Clinical Competence"[MeSH Terms] OR "Educational Measurement"[MeSH Terms] OR "Task Performance and Analysis"[MeSH Terms] OR "Surveys and Questionnaires"[MeSH Terms] OR "Clinical Competence"[Text Word] OR "Educational Measurement"[Text Word] OR "task performance"[Text Word] OR "task analysis"[Text Word] OR "survey*"[Text Word] OR "competency assessment"[Text Word] OR "technical skill assessment*"[Text Word] OR "technical skill score*"[Text Word] OR "tool*"[Text Word] OR "grading*"[Text Word] OR "checklist*"[Text Word] OR "check list*"[Text Word] OR "questionnaire*"[Text Word] OR "rating*"[Text Word] OR "scoring system"[Text Word] OR "classification system"[Text Word] OR "objective evaluation"[Text Word]) AND ("Colorectal Surgery"[MeSH Terms] OR "Colectomy"[MeSH Terms] OR "Colorectal Surgery"[Text Word] OR "Colectomy"[Text Word] OR "colon resection"[Text Word] OR "cme"[Text Word] OR "complete mesocolic excision"[Text Word] OR "lcme"[Text Word] OR "proctocolectomy"[Text Word] OR "colorectal surgeon*"[Text Word]) AND ("Laparoscopy"[MeSH Terms] OR "laparoscop*"[Text Word] OR "lcme"[Text Word]) |
| *The search strategy for Embase and PubMed/MEDLINE is reported as it was run in the database. Search 1 to 20 capture the concepts of ‘assessment’ and are combined using OR to produce the result search 21 for Embase, which equals the A search for PubMed/MEDLINE. Search 22 to 32 capture the concepts of ‘colon surgery’ and are combined using OR to produce the result 33 for Embase, which equals the B search for PubMed/MEDLINE. Search 31, 34 and 35 capture the concepts of ‘laparoscopy’ and are combined using OR to produce the result 36 for Embase, which equals search C for PubMed/MEDLINE. The three sets of concepts were then combined to identify the records which contain all three concepts using AND to produce the search 38 for Embase which equals the D search for PubMed/MEDLINE.* | |
